# Preparation of the Potential Ocular Inserts by Electrospinning Method to Achieve the Prolong Release Profile of Triamcinolone Acetonide

**DOI:** 10.15171/apb.2018.003

**Published:** 2018-03-18

**Authors:** Shahla Mirzaeei, Kaveh Berenjian, Rasol Khazaei

**Affiliations:** ^1^Pharmaceutical Sciences Research Center, School of Pharmacy, Kermanshah University of Medical Sciences, Kermanshah, Iran.; ^2^Nano Drug Delivery Research Center, School of Pharmacy, Kermanshah University of Medical Sciences, Kermanshah, Iran.; ^3^Student Research Committee, School of Pharmacy, Kermanshah University of Medical Sciences, Kermanshah, Iran.

**Keywords:** Ocular, Insert, Electrospinning, Nanofiber, Chitosan, Triamcinolone acetonide

## Abstract

***Purpose:*** The poor bioavailability of drugs in the ocular delivery systems is an important issue and development of delivery systems with prolonged release profile could be in a major importance. This study aims to develop an ocular delivery system using electrospun nanofibers to be a candidate insert for delivery of triamcinolone acetonide.

***Methods:*** For this purpose, three different chitosan-based formulations were prepared by electrospinning method, and electrospun nanofibers were compared to a formulation comprising hydrophobic polymers (Eudragit S100 and Zein). The electrospun nanofibers were characterized by SEM and FTIR analyses. The release profile and release kinetic models of all the formulations were also examined.

***Results:*** The SEM photographs of electrospun nanofibers revealed that among the four designed formulations, formulation obtained by electrospinning of chitosan and PVP possessed the best quality and the minimum size (120 ±30 nm) , which resulted the most uniform and bead-free nanofibers. This formulation also possessed the prolonged release profile of triamcinolone acetonide and was the only electrospun nanofiber following the zero-order kinetic profile. Due to the small diameter and uniformity of this formulation, the prolonged and well controlled release profile, it could be taken into account as a candidate to overcome the drawbacks of the commonly used ocular delivery systems and be used as ocular insert.

***Conclusion:*** This study confirmed the ability of electrospun nanofibers to be used as ocular inserts for delivery of ophthalmic drugs.

## Introduction

Topical formulations of corticosteroids, including drops and ointments, are the most prescribed formulations for treatment of different ocular disorders. Eye drops are the most frequently used ophthalmic drugs, due to the ease of instill and topical administration.^1^ This type of administration of ophthalmic drugs affects the anterior segment of the eye and is associated by significant drawbacks; the poor bioavailability of drug (due to nasolacrimal drainage and dilution of drug by tear turnover), requirement of frequent administration, unpredictable and uncontrolled doses, blurred vision, messy and difficult dosing, and short residence time are some of these drawbacks.^2-4^

Several efforts have been conducted to attain an effective formulation, with a desired concentration of drug at the intended site of action, for a programmed period of treatment.^5^ The therapeutic efficacy of an ocular drug can be greatly improved by prolonging its contact with the corneal surface.^6^

Ocular inserts are the solid and semi-solid drug-impregnated formulations which could be placed into cul-de-sac or conjunctiva sac.^6^ These inserts have been used frequently for reducing the frequency of administration, attaining the extended release duration, predictable and controlled release profile, and desired efficacy in treatment of eye infections such as conjunctivitis, keratitis, corneal ulcers.^3,7-10^

The main objective of this study is to produce the electrospun formulations which could be used as ophthalmic inserts and to incorporate triamcinolone acetonide into these formulations. These formulations hoped to be able to increase the contact time between the drug and conjunctival tissue, decrease the number of administration, achieve a controlled and prolonged release suited for topical treatment of infection, and improve the therapeutic efficacy of drug at the site of action.

Electrospinning is prevailing as a remarkably simple, well-established method which provides a versatile technique for generating various nanofibers with controlled surface morphology from different sources (polymers, ceramics, and composite).^11^ Electrospun fibers with different characteristics are promising scaffolds for drug encapsulation and cell incorporation and also can offer the cells an environment for mimicking the native extracellular matrix (ECM). These nano-scale scaffolds have been studied for adhesion, proliferation, and differentiation of different types of cells.^12^ Incorporation of bioactive agents into nanofibers can be occurred in one-step process with almost no loss of the agents. The high surface area to volume ratio of nanofibers, flexibility in surface functionalities,^13^ porous structure, and good mechanical strength make them promising candidates for many biomedical applications,^14-16^ such as controlling delivery of antibiotics and other antibacterial compounds.^17,18^ Natural polymers have been implicated as the opportunistic biomedical applications because of their biocompatibility, biodegradability and similarity to the ECM. The most frequently used natural polymers attracting much attention for the biomedical applications are the polysaccharides and proteins.^19^ Chitosan is a hydrophobic linear polysaccharide obtained from deacetylated chitin.^20^ Chitosan was chosen as the main polymer in this study because it is a water insoluble mucoadhesive polymer which can provide the prolong release of drugs and improve the ocular bioavailability of triamcinolone acetonide.^21,22^ Chitosan can interact chemically with the negatively charged mucus layer or the eye tissues and enhance the residence time of drug. ^3^ Based on the literature review, in spite of overwhelming popularity of electrospinning, no study has been devoted to the application of electrospinning in preparation of ocular inserts for delivery of ophthalmic therapeutics. Just besides of this study, one study investigated the application of electrospun PLA/PVA nanofibers as inserts in ocular delivery of dexamethasone.^23^ To the best of our knowledge, this is the first attempt towards preparing ocular inserts by chitosan-based electrospun nanofibers, to be used as the ocular inserts capable of controlling and prolonging the delivery of triamcinolone acetonide. In this study, electrospun chitosan-based nanofibers were fabricated to be used as ocular inserts. Furthermore, a formulation comprising two hydrophobic components (Eudragit/ Zein) was prepared and the results were compared to the chitosan-based nanofibers. Eudragit was chosen because it is suitable for loading the anionic drugs and also can attach to the negatively charged cells.^24^

## Materials and Methods

Chitosan and Zein were purchased from Sigma Aldrich (St. Louis, Missouri, USA). Eudragit S100 was procured from Evonik Industries, Parsippany, NJ, USA. Poly vinyl alcohol (PVA 13-23 kDa, 98% hydrolysis), poly vinyl pirolidine (PVP), and ethanol were procured from Merck (Germany). Triamcinolone acetonide was purchased from Crystal Pharma (Spain). All the other compounds were in analytical grade and were performed without further purification.

### Preparation of electrospinning solutions

In this study, four formulations were performed for producing triamcinolone acetonide-loaded nanofibers (Table 1). Chitosan-based electrospun nanofibers were obtained from the solution of chitosan in acetic acid. In order to prepare formulation T_2_, chitosan was dissolved in 1% acetic acid to obtain the concentration of 6% (w/v) and stirred for 4h at 300 rpm and PVA (12% w/v) was added to the solution. Formulation T_3_ comprised of chitosan, PVP, and PVA dissolving in water with concentrations of 6, 3, and 9%, respectively. The formulation T_3_ was obtained by co-dissolving chitosan (6% w/v) in acetic acid and addition of PVP (12% w/v) under stirring conditions at 300 rpm. The electrospinning solution of formulation T_1_ was prepared by dissolving Eudragit S100 in ethanol and Zein in isopropyl alcohol the solution was stirred for 5h. A drug solution with concentration of 1% w/v in acetic acid was added to each formulation.

**Table 1:** Different formulations prepared for electrospinning process, the arithmetic mean size and standard deviation of nanofibers.

**Formulation**	**PVA%**	**PVP%**	**Zein%**	**Eudragit%**	**Chitosan%**	Mean* size(nm)	**Standard Deviation(nm)**	**Viscosity (cps)**
T_1_	-	-	12.5	5	-	169	36	925
T_2_	-	12	-	-	6	120	30	1850
T_3_	9	3	-	-	6	260	73	1948
T_4_	12	-	-	-	6	172	48	1172

^*^ Arithmetic mean size of nanofibers

### Electrospinning

After preparation of electrospinning solutions, the solutions were placed in a 5 mL syringe connected with a metallic needle, with inner and outer diameters of 0.6 and 0.9 mm, at the nozzle. The solutions were pumped at the flow rate of 0.7 mL/h using a syringe pump (SP1000, fanavaran Nano-meghyas, Iran). The positive electrode of the Fanavaran Nano-meghyashigh-voltage power supply (D04X-05^TH^ , Fanavaran Nano-meghyas, Iran) was clamped to the tip of the needle and the electrospun nanofibers were collected on a grounded rotating cylindrical collector covered by aluminum foil. The needle tip to collector distance was fixed at 8 cm and the solutions were electrospun at a fixed voltage of 22 kV. The electrospinning of solutions were carried out at constant temperature of 40°C by controlling the temperature in a metallic box enclosing the electrospinning apparatus.

### Characterization of electrospun nanofibers

The diameter and morphology of electrospun nanofibers were analyzed by Scanning Electron Microscope (SEM, Hitachi, HT-4160-02, Japan). The nanofiber samples were placed on the aluminum stub and were gold coated prior to SEM imaging. The mean diameter of nanofibers was determined using ImageJ software. The diameter of at least 100 samples was measured and the arithmetic average diameter was obtained. The viscosity of electrospinning solutions was determined using BROOKFIELD ULTRA DV III Rheometer .

The structural change during electrospinning and the interaction of different chemical groups were observed by Fourier Transform Infrared (FTIR) spectroscopy using potassium bromide tabs. The range of spectra was in 400-4000 cm^-1^ with the accuracy of 4cm^-1^.

### Release study

The *in-vitro* release profile of triamcinolone acetonide from different formulations was determined to obtain the best formulation which is close to the aim of this study. The specified pieces of drug loaded electrospun nanofibers were placed in a dialysis bag (Mw cut-off= 12,000–14,000 Daltons; Delchimica Scientific Glassware, Milan, Italy), containing PBS (pH=7.4). The dialysis bag was sunk in PBS (pH=7.4) as release medium. The system was stirred continuously at 100 rpm and maintained at 37°C.At predetermined time intervals, a specified amount of release medium was replenished by fresh medium. The concentration of triamcinolone acetonide in withdrawn samples at different times was determined using UV spectrophotometer at 238 nm. This study was carried out in triplicate and the release curve of triamcinolone acetonide from each electrospun nanofiber was prepared.

## Results and Discussion

### Size and morphology of electrospun nanofibers

**Figure 1:**
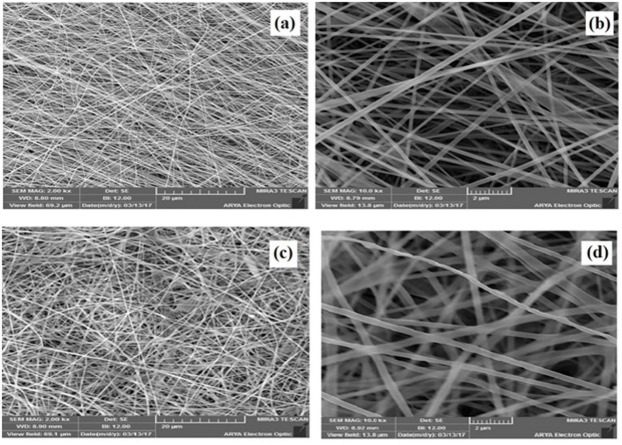
The SEM photographs of formulations (a) T_1_, (b) T_2_, (c) T_3_, and (d) T_4_.

The SEM photographs of the four electrospun nanofibers with different formulations are demonstrated in Figure 1 (a-d). The size and standard deviation of electrospun nanofibers were determined using ImageJ software, and the results were tabulated in Table 1. The SEM photographs indicated that formulations T_1_, T_2_, and T_4_ resulted in bead-free electrospun nanofibers with a smooth surface and uniform diameters, but in the formulation T_3,_ some scattered droplets were formed on the nanofiber and its beaded structure made formulation T_3_ unsuitable for medical applications. A glance at figure indicated that adding PVP to the structure of chitosan/PVA nanofibers caused bead formation and a decrease in the uniformity of nanofiber. The size of nanofibers was also compared and among the prepared formulations the best one with smallest diameter and standard deviation was formulation T_2_ (chitosan/ PVP) with the mean diameter of 120±30 nm. The viscosity of electrospinning solutions is also indexed in Table 1.

### FTIR results

**Figure 2:**
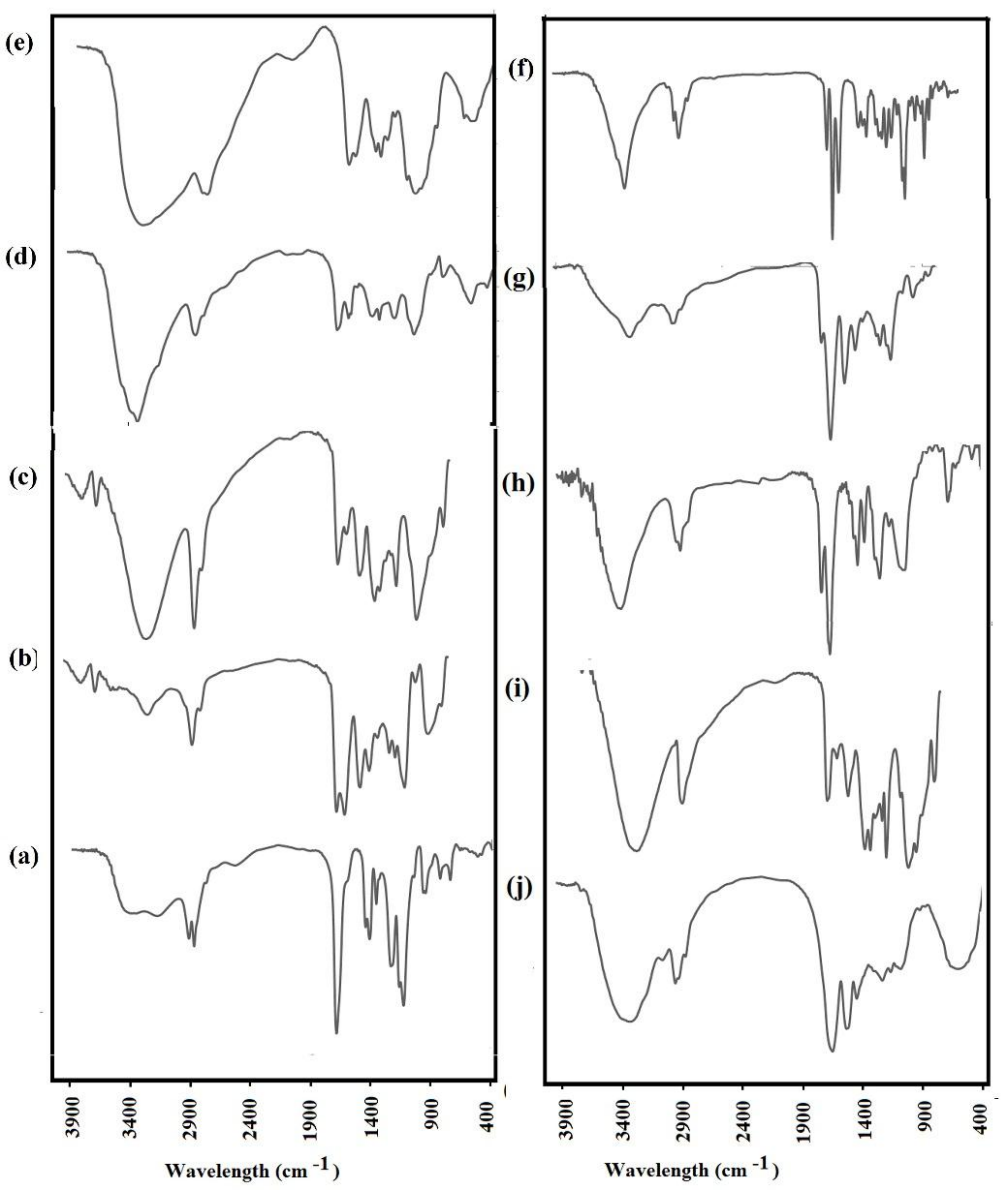
The FTIR spectra of pure Eudragit S100 (a), Zein (b), PVP (c), PVA (d), chitosan (e), triamcinolone acetonide (f), and electrospun nanofibers T_1_ (g), T_2_ (h), T_3_ (i) and T_4_ (k).

In drug delivery systems, it could be an important issue to find out the possible interactions of drug and polymer. The FTIR analysis could help the identification of these interactions based on the alteration of the spectra of the samples. In the present study, the FTIR spectra of pure polymers (Eudrsgit S100, Zein, chitosan, PVA, and PVA) and free drug were compared with the spectra of the four formulations. The FTIR spectrum of triamcinolone acetonide is demonstrated in Figure 2. FTIR spectrum of triamcinolone acetonide included the hydroxyl group stretching vibration at 3392 cm^-1^, the carbonyl group (C=O) band at 1708 cm^-1^, C=C stretching vibration band at 1662 cm^-1^, and C-O stretching vibration at 1207cm^-1^. In the spectrum of Eudragit S100, the characteristic peak at 1724 cm^-1^could be attributed to the esterified carbonyl group. Two peaks were observed at 1150, 1245cm^−1^ corresponding to the ester vibration. The broad peak from 3224 to 3452cm^−1^could be assigned to the absorption band of the hydroxyl group. The spectrum of Zein revealed characteristic peaks of amide I and amide II at 1651 and 1523 cm^-1^, which were respectively due to C=O stretching vibrations, N-H bending, and C-N stretching vibrations. The FTIR spectra of formulation T_1_ clarified that no significant interaction could be observed between the components of this formulation and drug because of the presence of major bands of components and drug, as well as the absence of new characteristic peaks. The typical infrared bands corresponding to the OH group of chitosan at 3410 cm^-1^, the NH_2_ group of chitosan at 1641 cm^-1^, and stretching band of the C-O functional group at 1365 cm^-1^ could be observed in the spectra of formulations T_2_, T_3_, and T_4_, while there were no significant shifting and alteration in the position of these peaks. The characteristic peaks of triamcinolone acetonide were identified in the spectra of these three formulations, at the same wavelengths (3292, 1708, 1600-1690, and 1234cm^-1^), compare to those of pure drug.

In the spectra of the formulation T_2_ andT_3_, the characteristic peaks of PVP ( 1716 cm^-1^ for C=O vibration, 1238 cm^-1^ corresponding to C-N vibration, and 3066-3522 cm^-1^corresponding to OH functional group ) and those corresponding to PVA (at 3296 cm^-1^ attributing to the OH vibration, the range of 2850-3000 representing the alkyl vibration) were observed without any alteration in the wavelength of the peaks.

The characteristic peaks of these three formulations seem to be the superposition of their components (polymers and drug) and the FTIR results of these formulations exhibited that drug has been physically dispersed into the polymeric chain, without the presence of detectable chemical interactions.

## Release study and release kinetic

In order to study the release pattern of triamcinolone acetonide from different electrospun nanofibers, the concentration of drug in release medium at specified time intervals were determined using a UV-Vis spectrophotometer. The absorbance band of solutions was recorded and the concentration of drug was calculated using the standard calibration curve, which correlated the concentration of triamcinolone acetonide to the absorbance intensity. The calibration curve followed the below equation:

y=0.035x+0.0038 R^2^ = 0.9998

Where y is the absorbance intensity at 238 nm and x is the concentration of triamcinolone acetonide in release medium.

**Figure 3:**
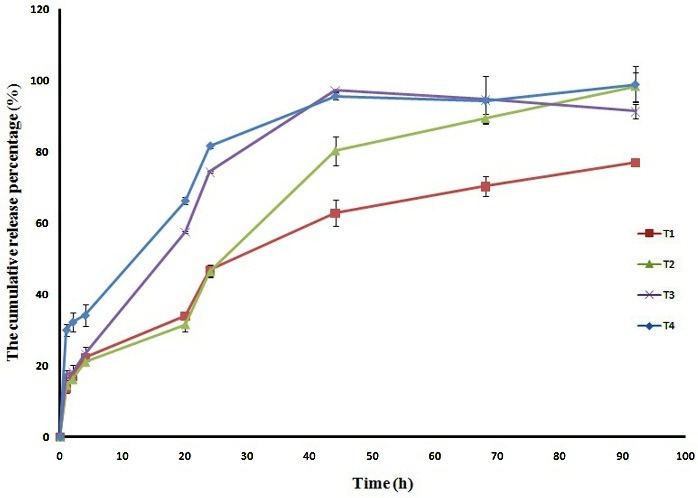
The release profile of triamcinolone acetonide from different electrospun nanofibers.

The release pattern of different electrospun nanofibers is represented in Figure 3.

A glance at figure indicated that triamcinolone acetonide released from these electrospun nanofibers T_1_, T_2_, and T_3_ without inducing burst release, but the formulation T_4_ released up to 30% of the loaded drug within the first hour. The absence of the burst release in the release profile of these nanofibers showed the potential of these formulations to control the release and delivery of triamcinolone acetonide. These formulations could be advantageous over the other forms of chitosan-based inserts because the release profile of drug contained burst release and those inserts were not able in controlling the release rate and delivery of drugs. According to the figure, the release profile of these formulated electrospun nanofibers was prolonged and these insert candidates released the drug for up to 4 days, while the release profile of free triamcinolone acetonide has been reported more than 70% within 8h.^24^ The slowest release rate was achieved for formulation T_1_, in which the EudragitS100 and Zein were performed in the electrospinning solution, following the formulation T_2_ (chitosan/PVP nanofiber). In order to compare the release rate of these formulations the time corresponding to 80% release of triamcinolone acetonide (t_80%_) was recorded. The t_80%_ was found up to 23, 25, and 44.8 h for formulations T_4_, T_3_, and T_2_ respectively, while formulation T_1_ did not release 80% of the loaded drug within the study period. The completed release of drug (about 99% release) was achieved for electrospun nanofiber T_2_ within 4 days, while the nanofiber T_1_ released about 77% of the loaded drug within this period. The slow release pattern of formulation T_1_ could be due to the hydrophobic nature of the polymers (Eudragit and Zein) and lower the dissolution rate of these polymers in release medium. The release profile of triamcinolone acetonide from Eudragit RS100 nanofibers with 20% polymer concentration showed a burst release within the first hour following the plateau release and 65% of drug released the nanofiber within 8h,^24^ while in the present study the release of 65% of loaded triamcinolone acetonide from Eudragit/Zein nanofibers took more than 70 h.

In order to perform a further comparison the triamcinolone acetonide release from different electrospun nanofibers, the kinetic study was conducted to find the best model correlating the release percentage to the time. The release data of each formulation were fitted to three commonly used empirical models (Higuchi, Zero-order, and First-order) and the amount of R^2^corresponding to each model was compared (Table 2). The kinetic study suggested that the Higuchi was the best fitting model for formulations T_1_, T_3_, and T_4_, while formulation T_2_ fitted to the Zero-order kinetic model. Zero-order kinetic with constant release rate and independent drug concentration was known as the ultimate goal of controlled-release delivery systems. The zero-order release profile of formulation T_2_, the small size, and size distribution of nanofibers suggested that this formulation is the closest one to the aim of this study.

**Table 2:** The R-squared of the fitted kinetic models for different formulations.

**Formulation**	**Higuchi**	**Zero-order**	**First-order**
**T**_1_	**0.9443**	0.8546	0.8861
**T**_2_	0.9074	**0.9461**	0.9060
**T**_3_	**0.9863**	0.9514	0.9337
**T**_4_	**0.9550**	0.8725	0.9313

The well-controlled and monotonic release profile of this formulation could be attributed to the monodispersity of the size of nanofibers. The nanofibers with different sizes released the drug with different rates, but the monodisperse nanofibers with homogenous sizes released the drug more predictably. This formulation was selected as the best electrospun nanofiber to be used as an ophthalmic insert. The release study also revealed the promising ability of Eudragit and Zein to prolong the release time of triamcinolone acetonide and achieve the sustained release, but based on the hydrophobic nature of the polymers, the swelling ability of this formulation in the cul-de-sac must be further investigated. It should be noted that although the release rate of the drug from hydrophobic formulation T_1_ was slower than formulation T_2_, the formulation T_2_ could be advantageous over T_1_. Based on the water solubility (and consequently the high swelling ability) of polymers and muco-adhesive nature of chitosan, the formulation T_2_ could be swelled in neutral pH of the lacrimal fluid and the solid inserts could be converted to a gel phase and could interact with mucus within muco-adhesion process.^3^ Therefore, the controlled drug lixiviation for a prolonged period could be achieved. While the hydrophobic nature of formulation T_1_ could possibly cause the degradation and erosion of the insert and dispersion into cul-de-sac, which might cause the problems such as blurred vision within the treatment period. Therefore the formulation T_2_ could be suggested for further investigations as a desired candidate for alternating the typical ocular delivery systems with ophthalmic inserts. This formulation also could be advantageous over the other ocular inserts suggested in the previous study, according to the monotonic, well-controlled, and well-prolonged release profile of triamcinolone acetonide.

The previous studies showed the lack of control over the release profile of ocular therapeutics from chitosan-based inserts (prepared by different methods), attributing the accumulation of drug on the surface of inserts and existence of burst release in their profile.^25-27^Jahangiri *et al*.^28^ used the electrospray method to prepare the triamcinolone acetonide-loaded PLGA nanobeads and nanofibers as the alternative delivery system for ophthalmic drops. In spite of the advantages of the prepared formulations as the ophthalmic inserts, the release profile included the initial burst release following the plateau release. Beside, the release period was much shorter than that of the electrospun formulations in this study. Prajapati et al. used the PVA/chitosan films as the ocular insert for prolonging the delivery of ofloxacin. The release of ofloxacin from the films was completed within the first 6h.^29^

The facility of the electrospun nanofibers administration on the ocular surface could be another important advantage of the suggested formulation. Furthermore, the other advantage of these inserts over the conventional ophthalmic delivery systems such as drops was that the multiple-dose typical ophthalmic delivery systems (such as drops) required and contained preservatives (such as benzalkonium chloride) in the formulation for protecting the therapeutics against the contamination by microorganisms.^3^ The presence of preservatives could mediate inflammation and corneal and conjunctival cell death,^29^ while these solid-state inserts did not require the addition of any preservative and could overcome the side effects corresponding to the presence of preservatives.

## Conclusion

For the first time in the present study, hydrophilic chitosan-based ocular inserts were developed for delivery of triamcinolone acetonide by electrospinning method and the results were compared with a hydrophobic (Eudragit/ Zein) electrospun insert candidate. The prepared ophthalmic delivery system could achieve prolonged therapeutic drug concentrations and could be promising candidates as ocular inserts and it could be used in a single dose at longer time intervals. The Zero-order release profile indicated the prolonged, monotonic, and well-controlled release of drug from the suggested formulation, and consequently, limited systemic exposure and side effects and the possibility to improve the patient adherence to therapy could be taken into account as some of the advantages of the suggested formulation. The suggested formulation could be converted from solid to gel form after exposition into the ocular pH, and the muco-adhesive nature of the suggested formulation caused the longer period for control of lixiviation than other similar formulations. Based on the advantages of the suggested formulation, it could be performed for ocular delivery of other similar drugs.

## Acknowledgments

The authors would like to acknowledge the Research Council of Kermanshah University of Medical Sciences (Grant number: 95416) for financial support of this work. Also faithfully thanks to Mr Komail Sadrjavadi and Miss Marziyeh Hajialyani for their assistances and valuable ideas.

## Ethical Issues

Not applicable.

## Conflict of Interests

The authors declared no conflict of interest for this study.
